# Fear–avoidance beliefs associated with perceived psychological and social factors at work among patients with neck and back pain: a cross-sectional multicentre study

**DOI:** 10.1186/1471-2474-14-329

**Published:** 2013-11-21

**Authors:** Kjersti Myhre, Cecilie Røe, Gunn Hege Marchand, Anne Keller, Erik Bautz-Holter, Gunnar Leivseth, Leiv Sandvik, Bjørn Lau

**Affiliations:** 1 Department of Physical Medicine and Rehabilitation, Oslo University Hospital, Ulleval, Oslo, Norway; 2 Faculty of Medicine, University of Oslo, Oslo, Norway; 3 Faculty of Medicine, Department of Neuroscience, Norwegian University of Science and Technology, Trondheim, Norway; 4 Department of Biostatistics and Epidemiology, Oslo University Hospital, Ulleval, Oslo, Norway; 5 National Institute of Occupational Health, Oslo, Norway; 6 Lovisenberg Hospital, Oslo, Norway

**Keywords:** Fear–avoidance beliefs, Neck pain, Back pain, Psychosocial work factors, Disability

## Abstract

**Background:**

Neck and back pain are common and often account for absenteeism at work. Factors at work as well as fear–avoidance beliefs may influence sick-leave in these patients. The aims of this study were to assess: (1) how sick-listed patients in specialised care perceive demand, control, support, effort, reward, and overcommitment at work compared to a general reference group of workers; (2) if women and men report demand, control, support, effort, reward, and overcommitment differently; and (3) the association between psychological and social factors at work and fear–avoidance beliefs about work.

**Methods:**

A cross-sectional multicentre study was carried out in 373 patients on sick leave due to neck and back pain. Psychosocial work factors were measured by demand, control, and support, (Nordic Questionnaire for Psychological and Social Factors at Work), and effort, reward and overcommitment (Effort Reward Imbalance Questionnaire). Fear avoidance beliefs about work were measured by the Fear–Avoidance Belief Questionnaire Work subscale (FABQ-W).

**Results:**

Although the patients differed significantly from a reference working group regarding several subscales of demand, control, support, effort, reward, and overcommitment, the magnitude of these differences were small. The study population also reported significantly higher scores for ‘demand for physical endurance’ than the reference population, and Cohen’s d = 0.55 here indicated a medium degree of difference. Female patients reported significantly higher on support, whereas male patients reported significantly higher demand for physical endurance, quantitative demand, effort, and overcommitment. Demand for physical endurance, job control, job support, high reward, and overcommitment were significantly associated with FABQ-W.

**Conclusions:**

Perceived psychological and social factors at work were strongly associated with fear–avoidance beliefs about work in sick-listed neck and back patients. The demand for physical endurance, control, support, high reward, as well as overcommittment at work outweighed pain and added to the burden of emotional distress and disability regarding fear–avoidance beliefs.

## Background

Neck and back disorders are common causes of pain and frequently lead to activity limitations and work absence. The total sickness benefit costs in Norway amounted to 36 billion Norwegian kroner (5 billion euros) in 2009. About 40% of the sick leave days were due to musculoskeletal disorders, with back pain as the predominant cause [1].

Although back pain is a benign condition in most subjects, 10% are not able to resume work after 3 months [2,3]. These subjects are often referred to specialised health care and are responsible for up to 90% of medical and compensation costs attributable to low back pain (LBP) [2,3]. Psychological distress and loss of function often accompany the pain [4]. Factors at work may also add to the disability [5]. Thus, when trying to understand the nature of sickness absence, we need also to look into the physical and psychosocial factors at work, in combination with the medical factors and the personal characteristics of each individual [6].

The demand–control model developed by Karasek and colleagues [7,8] is a 3D model integrating job demand, decision latitude, and social support at work. The model is based on research showing that workers with high-strain jobs and low social support have higher risk of cardiovascular disease. Later, this model was also used in research regarding occupational back pain [9-11], indicating increased risk for LBP with higher perceived work demands and lower supports [9]. The demand–control model does not take into account how the individual actually responds to the demand. In an attempt to demonstrate the role of individual coping strategies, Siegrist has introduced the effort–reward–imbalance (ERI) model [12]. This model assumes that high effort at work is exchanged by reward and that this reward is largely contributed by recognition, career opportunities and security at work. In this model, overcommitment describes the individual’s pattern of excessive work-related commitment together with a strong desire for approval and esteem [13]. Although overcommitment is considered as a psychological risk factor alone, the model claims a higher risk of reduced health in persons in whom ERI and overcommitment act together [14]. ERI and overcommitment are shown to be associated with self-reported poor health, musculoskeletal complaints, psychological distress, and work-related burnout [15,16]. Furthermore, ERI seems to increase the risk of LBP and neck pain related to work among vineyard employees [16]. The association between gender and the components in the demand-control and ERI model is reported differently [14,15,17]. However, the psychological and social factors at work in patient populations have seldom been investigated and compared with those in workers in general.

Among patients referred to specialised care with LBP, the impact of psychosocial factors has been extensively documented [18]. In these patients, beliefs about their LBP are also considered important. Lethem et al. [19] introduced the fear–avoidance model with fear of pain as the central concept. By linking fear–avoidance beliefs about work to work disability, Linton and Buer [20] stated that patients often associate their pain with work, and they found that fear–avoidance belief is an important predictor of sick leave. However, the perception of strain and burden of work may affect fear–avoidance beliefs about work in patients with LBP, and this issue has not previously been addressed.

The aims of this study were to assess: (1) how sick-listed patients in specialised care perceive demand, control, support, effort, reward, and overcommitment at work compared to a general reference group of workers; (2) if women and men report demand, control, support, effort, reward, and overcommitment differently; and (3) the association between psychological and social factors at work and fear–avoidance beliefs about work.

## Methods

### Design

This was a cross-sectional multicentre study of patients on sick leave due to neck and LBP. The study was conducted in accordance with the Helsinki Declaration. It was evaluated by the Regional Committees for Medical and Health Research Ethics in Southeast Norway (S09024b 2009/1000) and according to the Norwegian guidelines authorised by the Data Protection for Research at Oslo University Hospital (1207–091208).

### Participants

**Figure 1:**
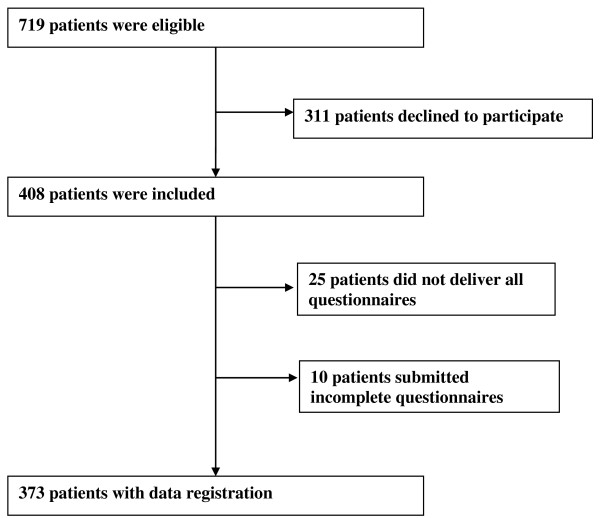
Flow chart of study.

We recruited patients referred to the neck and back outpatient clinic at Oslo University Hospital and St. Olavs University Hospital, Trondheim, Norway. Inclusion criteria were age 18–60 years, employed, and duration of sick leave between 4 weeks and 12 months. Exclusion criteria were patients in need of surgical treatment, cauda equina syndrome, and symptomatic spinal deformities, osteoporosis with fractures, inflammatory rheumatic diseases, other serious somatic or mental diseases, pregnancy, legal labour dispute, and insufficient Norwegian language to fill in the questionnaires. Between August 2009 and August 2011 a total of 3961 patients were screened for eligibility. The main reasons for ineligibility were: not sick-listed (50%); unemployed (26%); a disorder suitable for surgical treatment (7%); and lack of Norwegian language skills (6%). A total of 719 patients were eligible, and 408 of these gave their consent. A total of 31 included patients were removed from the analyses due to missing or incomplete scores on the Questionnaire for Psychological and Social Factors at Work (QPS) and ERI questionnaire, and 4 due to lacking responses in FABQ-W (Figure 1).

### Demographic factors and occupation

We recorded age, gender, native language, marital status, smoking status, highest level of education, and profession at baseline. Level of education was categorised into four groups: primary school, 7–10 years; vocational high school or general academic secondary school; college or university < 4 years; and college or university ≥4 years [21]. Occupations were categorised based on International Standard Classifications of Occupations, ISCO-88 [22]. Based on the ISCO-88 codes, we collapsed the occupations into four categories: low-skilled blue-collar workers (ISCO-codes 8 and 9); high-skilled blue-collar workers (ISCO-codes 6 and 7); low-skilled white-collar workers (ISCO-codes 4 and 5); and high-skilled white-collar workers (ISCO-codes 1, 2 and 3).

### Self-administered comorbidity questionnaire

A Norwegian version of the Self-Administered Comorbidity Questionnaire was used [23] to register 12 common comorbid conditions. We calculated the number of self-reported comorbid conditions in each patient.

### Pain

The participants reported pain in the neck/arm and back/leg during activity over the past week on an 11-point numeric rating scale ranging from 0 (no pain) to 10 (worst possible pain). Validity as evaluated to other pain measurements and reliability (ICC>0.8), have been documented previously [24]. In the analyses, the highest pain rating representing either neck/arm or back/leg pain was used. The pain distribution was reported in McGill pain drawing [25]. We divided the body into 10 regions and counted the number of marked regions in each patient [26].

### Functioning

Functioning was measured by the Norwegian version of the Oswestry Disability Index (ODI) and Neck Disability Index (NDI) in patients with LBP and neck pain, respectively. Previous studies have reported the validity and high reliability (evaluated by ICC) of ODI [27,28] and NDI [29]. ODI/NDI comprised 10 items categorised from 0 to 5, with higher ratings indicating increased levels of disability. The sum of the scores was presented as a percentage, where 0% represented no disability and 100% maximal disability. When a patient reported both on the NDI and the ODI questionnaire, the highest disability score was used as the patient’s score in a new variable. This variable was named Disability Index (DI) and used in the analyses.

### Fear–avoidance beliefs questionnaire

Waddell’s Fear–Avoidance Belief Questionnaire (FABQ) Norwegian version consists of two subscales, FABQ-W about work and FABQ-PA about physical activity. High reliability (ICC>0.8) and construct validity (Pearson correlation coefficient >0.6) have been documented previously [30,31]. The FABQ-W score is the sum of seven items. Each item is scored on a seven-point Likert scale from 0 (strongly disagree) to 6 (strongly agree), which gives a total range from 0 to 42. FABQ was developed to quantify the level of fear–avoidance beliefs. The items in the Fear Avoidance Beliefs Questionnaire about Work are presented in Table 1.

**Table 1:** Fear–Avoidance beliefs questionnaire about work

	**Completely**				**Completely**		
	**Disagree**		**Unsure**		**Agree**		
My pain was caused by my work or by an accident at work	0	1	2	3	4	5	6
My work aggravated my pain	0	1	2	3	4	5	6
My work is too heavy for me	0	1	2	3	4	5	6
My work makes or would make my pain worse	0	1	2	3	4	5	6
My work might harm my back	0	1	2	3	4	5	6
I should not do my normal work with my present pain	0	1	2	3	4	5	6
I do not think that I will be back to my normal work within 3 months	0	1	2	3	4	5	6

### Hopkins symptom checklist 10 (HSCL-10)

A short version of HSCL-10 was used to screen for psychological distress (depression and anxiety) during the previous 14 days. The 10 items were scored according to how much nuisance or inconvenience each symptom had caused, from 1 (not at all) to 4 (very much). The sum of the items divided by the number of items was calculated and reported. The reliability (Cronbach alpha = 0.88) and validity as evaluated by high correlation to mental health measurements, have been reported previously [32,33].

### Nordic questionnaire for psychological and social factors at work (QPS Nordic)

QPS Nordic [34] is a questionnaire to identify psychological and social factors at work. The validity and reliability have been documented previously [17]. It was constructed on the basis of common international questionnaires on this subject, including the Job Content Questionnaire [35]. The total questionnaire comprises questions that are found to be important for health and well-being, independent of specific models. The QPS Nordic items covering the dimensions demand, control, and social support were used in this study. An overview of the subscales and items studied in these analyses are given in Table 2. In the regression analyses, a composite score for job demands, job control, and job support based on the sum of respective subscales was used.

**Table 2:** Overview of subscales and items from QPS Nordic used in the analyses

**Composite subscale**	**Subscales**	**Number of items**	**Total range of scores**
Demand	Quantitative demands	4 items	1–5*
	Control demands	3 items	1–5*
	Learning demands	3 items	1–5*
Control	Positive challenge at work	3 items	1–5*
	Control of decision	5 items	1–5*
	Control of work pacing	4 items	1–5*
Support	Support from superior	3 items	1–5*
	Support from co-workers	2 items	1–5*
	Support from friends and family	3 items	1–5*
Single item	‘Does your work require physical endurance’?	Single item	1–5*

*Responses were given along a five-point scale ranging from 1 (very seldom or never) to 5 (very often or always). For each subscale, we reported the sum of the item score divided by the number of items (range 1–5).

### ERI questionnaire

Psychosocial work factors according to the ERI model were obtained by using the Norwegian version of the ERI questionnaire. The evidence of validity and reliability has been documented previously [14,15]. Table 3 shows a list of included subscales and associated range of scores.

**Table 3:** Overview of subscales and response options in ERI Questionnaire

**Subscale**	**Number of items**	**Response options**	**Total range of scores**
Effort	5 items	1 (does not apply)	5–25*
		2 (does apply, but not strained)	
		3 (does apply and somewhat strained)	
		4 (does apply and strained)	
		5 (does apply and very strained)	
Reward	11 items	1 (does apply)	11–55^†^
		2 (does not apply, but not strained)	
		3 (does not apply and somewhat strained)	
		4 (does not apply and strained)	
		5 (does not apply and very strained)	
Overcommitment	6 items	1 (strongly disagree)	6–24^‡^
		2 (disagree)	
		3 (agree)	
		4 (strongly agree)	

*High score representing high effort with strain.

^†^Low score representing low reward with strain.

^‡^High score representing high level of overcommitment.

### Reference sample characteristics

The reference groups for QPS Nordic subscales were obtained from the population used when constructing and validating the questionnaire [17]. It consisted of 64% women, the mean age was 43.5 years, 5% were >60 years, and 53% had a college or higher level of education. The reference group for ERI subscales was a collection of employees in a municipality used to validate a Norwegian version of the ERI questionnaire [15]. In this population, 80% were women, median age 40–49 years, with 14% >60 years, and 94% were white-collar workers.

### Data analysis and statistics

We compared the average subscales values of the study population with the QPS reference population comprising 2010 employees from the four Nordic countries, from production industries, private service companies, health sector, and public administration [34], and the ERI reference population comprising 1803 employees in a medium-sized Norwegian municipality [15]. *t* tests were used to compare the study population with the reference population and to compare differences between men and women regarding the QPS and ERI subscales. To assess the size of the differences, we calculated Cohen’s d values [36]. Cohen’s d is defined as the difference between two means divided by the pooled standard deviation. We used the definition of effect sizes as given by Cohen; small (d = 0.2), medium (d = 0.5) and large (d = 0.8).

Hierarchical multiple regression analyses were performed to explore to what extent psychological and social work factors were related to FABQ-W. Missing values were treated as follows: if >5 items were missing, no total ODI/NDI scores were calculated. One or two missing items in HSCL-10 were substituted by the subject’s mean value. If more items were missing, the total HSCL-10 score was substituted by the mean value of the patient group, which was done with six participants. QPS and ERI scale scores were calculated as mean scores of completed items for those completing at least two-thirds of the corresponding items. In eight subjects more than one-third of the items in a subscale were missing. In order not to lose the information from these patients on other subscales, the total subscale was substituted by the mean value of the patient group. Subsequently, the calculations were performed in two steps. First, we divided possible independent variables into three boxes: demographic, relevant clinical variables, and psychosocial work factors. In each box, a standard regression analysis was performed, with variables entered one at a time. Variables with p values < 0.2 were finally included in a multivariate analysis. Age, gender, and other significant demographic variables were controlled for in all boxes. In the demographic box, educational level was collapsed into two categories based on FABQ-W distribution: those with or without a college/university degree. In the same way, the distribution of FABQ-W between the four occupational categories let us merge the two blue-collar categories into one. In the clinical box, pain, DI, HSCL-10, number of painful body regions, and number of comorbid conditions were included. The HSCL-10 was categorised into quartiles to examine the FABQ-W distribution. This exploration showed a linear variability of FABQ-W, and consequently, we kept HSCL-10 as a continuous variable. In the box with psychological and social work factors, job demands, job control, job support, effort, reward, overcommitment, and the single QPS item ‘Does your work require physical endurance?’ were included. The last item accounted for the physical burden at the work site. The response to this item was dichotomised into yes (4 or 5) or no (1, 2 or 3). Each variable was assessed with respect to normal distribution. Owing to a highly skewed distribution, ‘reward’ was categorised into quartiles. Otherwise, the remaining variables were regarded as normally distributed and hence were kept as continuous variables.

Second, all variables with p < 0.2 from previous multivariate analyses were included in the final multiple regression analysis. Low colinearity was found between the independent variables. The R^2^ value was reported for each step. In the final model, a statistical significance level of p < 0.05 was adopted. Statistical analyses were performed using PASW Statistics, version 18 (SPSS Inc., Chicago IL, USA).

## Results

The analyses were performed with full data registration from 373 patients.

### Patient characteristics

Demographic characteristics of the patients are reported in Table 4. Forty per cent of the patients reported at least one comorbid condition, with depression being the most frequent.

**Table 4:** Baseline characteristics of participants

**Variables**	
Age (yr) (mean, SD), n = 373	40.9 (9.8)
Female gender (n, %), n = 373	173 (46.4)
Education level (n, %), n = 373	
Primary school	58 (15.5)
Vocational high school/general secondary school	212 (56.8)
College/university < 4 years	62 (16.6)
College/university > 4 years	41 (11.0)
Occupational categories (n, %), n = 373	
Low-skilled blue-collar	61 (16.4)
High-skilled blue-collar	84 (22.5)
Low-skilled white-collar	126 (33.8)
High-skilled white-collar	102 (27.3)
Pain location (n, %), n = 373	
Neck	32 (8.6)
Neck and back	106 (28.4)
Back	235 (63.0)
Pain intensity at rest (range 0–10) (mean, SD), n = 373	4.7 (2.3)
Pain intensity on activity (range 0–10) (mean, SD), n = 373	6.2 (2.2)
Number of pain regions (range 0–10) (mean, SD), n = 372	3.5 (1.9)
Oswestry disability index (range 0–100) (mean, SD), n = 348	35.4 (13.3)
Neck disability index (range 0–100) (mean, SD), n = 165	38.1 (14.6)
Disability Index (range 0–100) (mean, SD), n = 373	38.4 (13.4)
Hopkins symptom checklist 10 (range 1–4) (mean, SD), n = 373	2.0 (0.6)
Fear-avoidance beliefs questionnaire, physical activity (range 0–24) (mean, SD), n = 369	13.6 (5.6)
Fear-avoidance beliefs questionnaire, work (range 0–42) (mean, SD), n = 373	27.5 (10.2)

Baseline characteristics of the participants (n = 373) among patients on sick leave due to neck or back pain and referred to specialised health care, given as mean (±SD) or number (%).

*SD* = Standard Deviation.

Fifty-three per cent of the patients reported to have been on sick leave for >100 days at the time of inclusion, yet 95% believed that in 2 years they would have returned to work. The age and gender distributions among consenters were similar to those of the neck and back outpatient population.

### Demand–control–support and effort–reward–overcommitment

The included patients differed significantly from the reference group with perceived lower quantitative and learning demands, lower control of decision and work pacing, and lower support from co-workers (p = 0.006). However, the magnitude of the difference was small (Cohen’s d < 0.23). We found a significant difference for the single item ‘Does your work require physical endurance?’ (p < 0.001) and a medium effect size (Cohen’s d = 0.55), supporting a higher reported physical endurance requirement by the study population compared to the reference group. Also, the study population reported significant higher effort and overcommitment, and lower reward than the reference population (p < 0.001), but the effect sizes were small (Cohen’s d between -0.26 and +0.34) (Table 5).

**Table 5:** **Two-sample independent *t *** **test and Cohen’s d for comparison of perceived psychological and social factors at work as measured by QPS Nordic and ERI Questionnaire subscales, for the study population of patients on sick leave due to neck or back pain, and a reference population**

**Subscales**	**Study population**		**Reference population**		**Cohen’s d**	***t*** **test**
						**p value**
**QPS Nordic**	Mean	SD	Mean	SD		
Job demands	n = 373		n = 2015			
Quantitative demands	3.14	0.82	3.26	0.77	-0.15	0.006*
Decision demands	3.49	0.82	3.55	0.77	-0.08	0.17
Learning demands	2.47	0.68	2.63	0.71	-0.23	< 0.001*
Job control						
Positive challenge at work	3.89	0.88	3.94	0.83	-0.06	0.29
Control of decision	2.62	0.85	2.76	0.82	-0.17	0.002*
Control of work pacing	2.57	1.14	2.81	1.18	-0.21	< 0.001*
Job support						
Support from superior	3.48	1.07	3.49	1.00	-0.01	0.86
Support from coworkers	3.66	1.03	3.88	0.89	-0.23	< 0.001*
Support from friends	3.92	0.99	3.91	0.92	0.01	0.85
Does your work require physical endurance?	3.44	1.42	2.70	1.30	0.55	< 0.001*
**ERI-Q**			n = 1803	SE		
Mean effort	12.82	4.53	11.7	4.2	0.26	< 0.001*
Mean reward	45.39	8.97	47.8	6.5	-0.31	< 0.001*
Mean overcommitment	13.32	3.76	12.1	3.4	0.34	< 0.001*

Mean values, standard deviation, Cohen’s d and *t* test of the QPS Nordic and ERI subscales.

*p < 0.05.

*SD* Standard Deviation, *CI* Confidence Interval.

Female patients reported significantly higher support from co-workers (3.88 vs. 3.48, p < 0.001), and support from friends and family (4.06 vs. 3.80, p = 0.01) compared with male patients. Male patients, however, reported a significantly higher demand for physical endurance than female patients (3.64 vs. 3.23, p = 0.002). They also reported significantly higher quantitative demand (p = 0.045), higher effort (p = 0.02), and higher overcommitment (p = 0.02) than female patients.

### Influence of demand–control–support and effort–reward–overcommitment on fear– avoidance beliefs about work

The results from the regression analyses in each box are presented in Tables 6, 7, 8. The result from the hierarchical multiple regression analysis is presented in Table 9. In Step 1, increasing age, and having a high-skilled white-collar occupation were significantly associated with lower FABQ-W score, whereas being a man was significantly associated with higher FABQ-W score in the multivariate model (Table 9). This model explained only 9% of the variability of FABQ-W. In Step 2, only DI and HSCL-10 from the clinical box remained significantly associated with FABQ-W, with increasing disability and emotional distress scores indicating higher FABQ-W scores. This step explained an additional 9% of the variability (Table 9). In Step 3, decreasing job control, increasing job support, increasing demand for physical endurance, and increasing overcommitment were significantly associated with increasing FABQ-W score. Furthermore, the two upper reward categories (3 and 4) were significantly associated with decreasing FABQ-W scores. In the final regression model, 39% of the variability in FABQ-W was explained (Table 9). Inclusion of the psychological and social factors at work increased the explained variance by 20%.

**Table 6:** Univariate and multivariate regression analysis with demographic factors as independent variables and FABQ-W as the dependent variable in sick-listed patients with neck or back pain

	**Univariate analysis**			**Multivariate analysis**		
**Independent variables**	**β**	**95% CI for β**	**p value**	**β**	**95% CI for β**	**p value**
Age (increase of 9.8 yr.)*	-1.44	-2.46 to -0.41	0.006	-1.15	-2.15 to -0.15	0.025
Gender (men vs. women)	4.20	2.17–6.23	< 0.001	3.02	0.93–5.10	0.005
Education (high vs. low education)	-3.99	-6.27 to -1.71	0.001	-0.96	-3.70 to 1.77	0.49
Low-skilled white-collar (vs. blue-collar)	-0.86	-3.05 to 1.33	0.44			
High-skilled white-collar (vs. blue-collar)	-5.15	-7.41 to -2.89	< 0.001	-3.39	-6.16 to -0.62	0.016

Both univariate and multivariate regression coefficients are given. Only independent variables with p < 0.2 in the univariate analyses are included in multivariate analysis.

*For continuous variables, β coefficient is given for an increase in the variable of 1 SD. *SD* Standard Deviation, *CI* Confidence Interval.

**Table 7:** Univariate and multivariate regression analysis with pain, disability (DI), emotional distress (HSCL-10), pain distribution, and comorbidity as predictors and FABQ-W as the dependent variable in sick-listed patients with neck or back pain, controlling for age, gender and occupation

	**Univariate**			**Multivariate**		
**Independent variables**	**β**	**95% CI for β**	**p value**	**β**	**95% CI for β**	**p value**
Age (increase of 9.8 yr)*				-1.16	-2.15 to -0.17	0.022
Gender (men vs. women)				3.66	1.67–5.65	< 0.001
High-skilled white-collar (vs. blue-collar)				-3.67	-5.89 to -1.44	0.001
Pain (increase of 2.21 pts)*	1.24	0.21–2.27	0.019	-0.17	-1.22 to 0.87	0.74
DI (increase of 13.4 pts)*	1.95	0.94–2.97	< 0.001	1.11	-0.03 to 2.25	0.057
HSCL-10 (increase of 0.57 pts)*	2.97	1.98–3.96	< 0.001	2.32	1.20–3.43	< 0.001
Number of pain regions (increase of 1.93 regions)*	0.08	-0.96 to 1.12	0.88			
Number of comorbid conditions (increase of 0.83 conditions)*	0.98	-0.05 to 2.01	0.063	0.50	-0.52 to 1.52	0.33

Both univariate and multivariate regression coefficients are given. Only independent variables with p < 0.2 in the univariate analyses are included in multivariate analysis.

*For continuous variables, β coefficient is given for an increase in the variable of 1 SD.

*SD* Standard Deviation, *CI* Confidence Interval.

**Table 8:** Univariate and multivariate regression analysis with perceived psychological and social factors at work as measured by QPS Nordic and ERI Questionnaire subscales as predictors and FABQ-W as the dependent variable in sick-listed patients with neck or back pain, controlling for age, gender and occupation

	**Unadjusted**			**Adjusted**		
**Independent variables**	**β**	**95% CI for β**	**p value**	**β**	**95% CI for β**	**p value**
Age (increase of 9.8 yr)*				-0.55	-1.43 to 0.33	0.22
Gender (men vs. women)				2.23	0.42–4.04	0.016
High-skilled white-collar (vs. blue-collar)				-2.31	-4.44 to -0.19	0.033
Job Demand (increase of 1.80 pts)*	2.97	1.98–3.96	< 0.001	0.17	-1.04 to 1.38	0.78
Job control (increase of 2.16 pts)*	-2.75	-3.74 to -1.75	< 0.001	-1.50	-2.49 to -0.50	0.003
Job support (increase of 2.48 pts)*	-1.50	-2.53 to -0.48	0.004	1.26	0.24–2.72	0.015
Demand for physical endurance (vs. seldom or never)	8.74	6.86–10.63	< 0.001	6.22	4.34–8.10	< 0.001
Effort (increase of 4.53 pts)*	3.05	2.06–4.04	< 0.001	0.65	-0.60 to 1.89	0.31
Reward cat.2 (vs. reward cat. 1)	1.35	-1.02 to 3.72	0.26			
Reward cat. 3 (vs. reward cat. 1)	-2.75	-5.06 to -0.43	0.02	-3.08	-5.22 to -0.94	0.005
Reward cat. 4 (vs. reward cat. 1)	-5.60	-8.02 to -3.17	< 0.001	-4.56	-7.06 to -2.06	< 0.001
Overcommitment (increase of 3.76 pts)*	3.43	2.46–4.41	< 0.001	2.34	1.26–3.42	< 0.001

Both univariate and multivariate regression coefficients are given. Only independent variables with p < 0.2 in the univariate analyses are included in multivariate analysis.

*For continuous variables, β coefficient is given for an increase in the variable of 1 SD.

*SD* Standard Deviation, *CI* Confidence Interval.

**Table 9:** Stepwise multiple regression analysis with disability (DI), emotional distress (HSCL-10), and perceived psychological and social factors at work as predictors and FABQ-W as the dependent variable in sick-listed patients with neck or back pain, controlling for age, gender and occupation

**Step**	**Independent variables**	**β**	**95% CI for β**	**p value**	**R**^**2**^**(%)**
1	Age (increase of 9.8 yr)†	-1.15	-2.15 to -0.15	0.02*	9
	Gender (men vs. women)	3.11	1.04–5.18	0.003*	
	High-skilled white-collar (vs. blue-collar)	-3.92	-6.24 to -1.59	0.001*	
2	Age (increase of 9.8 yr)†	-1.03	-1.98 to -0.08	0.03*	18
	Gender (men vs. women)	3.64	1.67–5.62	< 0.001*	
	High-skilled white-collar (vs. blue-collar)	-3.68	-5.89 to -1.46	0.001*	
	DI (increase of 13.4 pts)†	1.09	0.001–2.17	0.05*	
	HSCL-10 (increase of 0.57 pts)†	2.41	1.33–3.49	< 0.001*	
3	Age (increase of 9.8 y.)†	-0.46	-1.32 to 0.40	0.29	39
	Gender (men vs. women)	2.94	1.14 to 4.74	0.001*	
	High-skilled white-collar (vs. blue-collar)	-1.98	-4.05 – 0.10	0.06	
	DI (increase of 13.4 pts)†	1.10	0.15–2.05	0.02*	
	HSCL-10 (increase of 0.57 pts)†	1.06	0.06–2.07	0.04*	
	Job control (increase of 2.16 pts)†	-1.48	-2.45 to -0.50	0.003*	
	Job support (increase of 2.48 pts)†	1.45	0.45–2.45	0.005*	
	Demand for physical endurance (vs. seldom or never)	6.49	4.73–8.25	< 0.001*	
	Job reward quartile 3 (vs. reward cat. 1)	-2.98	-5.03 to -0.93	0.004*	
	Job reward quartile 4 (vs. reward cat. 1)	-4.48	-6.91 to -2.05	< 0.001*	
	Overcommitment (increase of 3.76 pts)†	2.13	1.19–3.07	< 0.001*	

Variables with p < 0.2 from previous multivariate analyses are included. The perceived psychological and social factors at work are measured by QPS Nordic and ERI Questionnaire subscales.

Adjusted regression coefficients, β, and R^2^ are given.

*p < 0.05.

†For continuous variables, β coefficient is given for an increase in the variable of 1 SD.

*SD* Standard Deviation, *CI* Confidence Interval.

Similar results were obtained when analysing men and women separately, except for DI which was a predictor for FABQ-W only for men (β = 1.83, p = 0.007).

## Discussion

In this study, the psychological and social work factors were significantly and relatively strongly associated with fear–avoidance beliefs about work. These factors, in addition to disability and psychological distress, explained 39% of fear–avoidance beliefs in patients on sick leave due to neck or back pain.

Description of psychological and social factors at work and possible association with neck or back pain, have mainly been studied in worker populations [9-11,16,37-39]. In a review from 2004, Hartvigsen et al. [37] concluded that LBP was not significantly associated with demand and control. Furthermore, moderate evidence for no association between social support and LBP is reported. However, later studies have shown associations between psychological and social factors at work and neck and back pain [9-11,16,38-40]. The present population reported a lower level of most aspects of demand, control, support, and reward, whereas effort and overcommitment were reported at a higher level than the reference population, although the differences were small [15,34]. The relationship between psychological and social factors at work and pain may change with duration of disability and transition into patient status. The variety of work places among the present patients may also influence the results. The perceived higher support from co-workers and family and friends by female patients are consistent with data in the reference population. However, the higher quantitative demands, effort and overcommitment as reported by men than women, are not consistent with reference population.

The study population reported higher demand for physical endurance than the reference population. It is well known that physical work demands are associated with LBP prevalence in specific occupational populations [10,11,41] and in the general worker population [42]. In our population, we did not have information about the physical workload to which it was actually exposed. Nevertheless, half of the population reported a demand for physical endurance. It may be that being troubled by pain makes one perceive the work situation to be more physically demanding than usual.

The mean pain score of 5–6 in the study population was comparable to that in other studies on sick-listed workers with chronic LBP in secondary care [31,43-45]. Computer workers with neck pain, but not sick-listed [46], have reported a lower pain level than in our population, whereas neck pain patients with more permanent work disabilities were characterised by even higher pain level than in the present study [47]. The average ODI score reported in the present study was slightly higher than that reported in primary care populations [4,48] and slightly lower than in patients recruited from secondary care populations [4,28]. However, our inclusion criteria demanding duration of sick leave < 1 year may have rendered us with a slightly less chronic LBP cohort.

Psychological distress in our study population, reported by HSCL-10, was much higher than in the general population [32]. However, a similar level of psychological distress has been reported by Brox et al. [4] in chronic LBP. In our population, 54% of the patients reported values above the recommended cut-off level, which indicates experience of significant psychological distress.

To the best of our knowledge, the impact of psychosocial work factors on fear–avoidance beliefs has not been evaluated previously. The factors underlying fear–avoidance beliefs are important to capture because these beliefs are a major predictor of work loss and disability [30,31,49-52]. It is well known that medical factors such as pain and disability, along with more personal factors such as depressive symptoms and anxiety, are associated with fear–avoidance beliefs [6,30,31]. We also know that perceived psychosocial factors at work are closely associated with anxiety and depression [53,54]. However, in our study, emotional distress continued to make a unique contribution to fear–avoidance beliefs, in addition to psychosocial factors at work. Similarly, our analysis showed that both gender and disability still provide their own contribution to fear–avoidance beliefs about work. The association with disability is generally known, whereas the association with gender varies between studies [30,31]. In our model, pain did not contribute to fear–avoidance beliefs. This is consistent with other studies that found low or no correlation between fear–avoidance beliefs about work and pain intensity [30,31]. Our findings emphasise the importance of identifying psychological and social work factors and including them in the assessment of prognosis for recovery or work loss, in addition to medical and emotional factors.

### Limitations and strengths

The present cohort was recruited from individuals in specialised care, and selected regarding language skills. The similar age and gender distributions among the consenters and non-consenters and the screening of all referred patients for eligibility precluded a representative patient cohort. However, these patients had a wide variety of occupations, which may have concealed potential differences from the reference population. The perceived burden of work may have been influenced by LBP. Furthermore, the lack of more objective assessment of exposure was a limitation, along with most studies conducted in this field [55].

The reference populations had a greater proportion of women and greater proportions with higher educational level or white-collar workers than the study population. This may have contributed to the difference regarding perception of demands for physical endurance.

The regression analyses were performed with women and men together, and this may have concealed different associations for men and women. Although performing a stratified analysis resulted in reduction of power, we clearly saw that disability was of significance for men only. None of the significant associations showed diverging directions for men compared with women in this analysis.

The use of a cross-sectional study design limited the analyses to explore associations, and not to draw any inference of causality in the associations found. The results imply a focus on the social and psychological factors at work in treatment and rehabilitation. However, as the actual prognostic value of the demand, control, and support in work for return to work in this patient population could not be established due to the cross-sectional design, a prospective study would be preferable as a basis for advices of implementation.

## Conclusion

Our study population of sick-listed neck and back pain patients reported mostly significant differences in average perception of demand, control, support, effort, reward, and overcommitment at work than workers in general, however, the differences were small. Perceived lower job control, higher job support, higher demand for physical endurance, and higher overcommitment were strongly associated with higher fear–avoidance beliefs about work in sick-listed neck and back pain patients. However, perception of higher reward was associated with lower fear–avoidance beliefs about work. The work-related factors outweighed pain and added to the burden of emotional distress and disability regarding fear–avoidance beliefs. The present study emphasises the need to focus on the work-related factors in sick-listed patients in specialised care.

## Abbreviations

DI: Disability index; ERI: Effort–reward imbalance questionnaire; FABQ: Fear–avoidance belief questionnaire; FABQ-W: Fear–avoidance belief questionnaire about work; HSCL-10: Hopkins symptom checklist 10; LBP: Low back pain; NDI: Neck disability index; ODI: Oswestry disability index; QPS: Nordic questionnaire for psychological and social factors at work.

## Competing interests

The authors declare that they have no competing interests.

## Authors’ contributions

KM participated in the design and coordination of the study, data acquisition, statistical analysis and interpretation of data, and drafting the manuscript. CR participated in the conception, design, and coordination of the study, analysis and interpretation of data, and critical revision of the manuscript. GHM participated in the design and coordination of the study, data acquisition, and critical revision of the manuscript. AK participated in the design and conception of the study, analysis and interpretation of data, and critical revision of the manuscript. EBH participated in the design and conception of the study, interpretation of data, and critical revision of the manuscript. GL participated in the design of the study, interpretation of data, and critical revision of the manuscript. LS participated in the statistical analysis and interpretation of data, and critical revision of the manuscript. BL participated in the conception and design of the study, analysis and interpretation of data, and critical revision of the manuscript. All authors read and approved the manuscript.

## Pre-publication history

The pre-publication history for this paper can be accessed here:

http://www.biomedcentral.com/1471-2474/14/329/prepub
